# 2-Oxo-2*H*-chromen-7-yl 4-*tert*-butyl­benzoate

**DOI:** 10.1107/S2056989018004188

**Published:** 2018-03-16

**Authors:** Mohammad Ouédraogo, Akoun Abou, Abdoulaye Djandé, Olivier Ouari, T. Jérémie Zoueu

**Affiliations:** a Laboratoire de Chimie Moléculaire et de Matériaux (LCMM), Equipe de Chimie Organique et de Phytochimie, Université Ouaga I Pr Joseph KI-ZERBO, 03 BP 7021 Ouagadougou 03, Burkina Faso; b Unité Mixte de Recherche et d’Innovation en Electronique et d’Electricité Appliquées (UMRI EEA), Equipe de Recherche: Instrumentation Image et Spectroscopie (L2IS), DFR–GEE, Institut National Polytechnique Félix Houphouët-Boigny (INPHB), BP 1093, Yamoussoukro, Côte d’Ivoire; c Institut de Chimie Radicalaire, Equipe SREP, UMR 7273 Aix-Marseille Université, Avenue Escadrille Normandie-Niemen, Service 521, 13397 Marseille cedex 20, France

**Keywords:** crystal structure, C—H⋯O hydrogen bond, coumarin, Hirshfeld surface analysis, quantum chemical calculations

## Abstract

In the title compound, C_20_H_18_O_4_, the benzoate ring is oriented at an acute angle of 33.10 (12)° with respect to the planar (r.m.s deviation = 0.016 Å) coumarin ring system. An intra­molecular C—H⋯O hydrogen bond closes an *S*(6) ring motif. In the crystal, C—H⋯O contacts generate infinite *C*(6) chains along the *b*-axis direction. Also present are π–π stacking inter­actions between neighbouring pyrone and benzene rings [centroid–centroid distance = 3.7034 (18) Å] and C=O⋯π inter­actions [O⋯centroid = 3.760 (3) Å]. The data obtained from quantum chemical calculations performed on the title compound are in good agreement with the observed structure, although the calculated C—O—C—C torsion angle between the coumarin ring system and the benzoate ring (129.1°) is somewhat lower than the observed value [141.3 (3)°]. Hirshfeld surface analysis has been used to confirm and qu­antify the supra­molecular inter­actions.

## Abstract 2

The structure of a coumarin ester is reported and compared with the results of a quantum chemical calculation. In the crystal, inter­molecular C—H⋯O contacts generate an infinite C(6) chain along the *b* axis. C=O⋯π and π–π stacking inter­actions also occur. Hirshfeld surface analysis was used to confirm and qu­antify the supra­molecular inter­actions.

## Chemical context   

Coumarins and their derivatives constitute one of the major classes of naturally occurring compounds and inter­est in their chemistry continues unabated because of their usefulness as biologically active agents. They also form the *core* of several mol­ecules of pharmaceutical importance. Coumarin and its derivatives have been reported to serve as anti-bacterial (Basanagouda *et al.*, 2009 ▸), anti-oxidant (Vukovic *et al.*, 2010 ▸) and anti-inflammatory agents (Emmanuel-Giota *et al.*, 2001 ▸). In view of their importance and as a continuation of our work on the crystal structure analysis of coumarin derivatives (Abou *et al.*, 2012 ▸, 2013 ▸), we report herein the synthesis, crystal structure, geometry optimization and Hirshfeld surface analysis of the title coumarin derivative, (I).
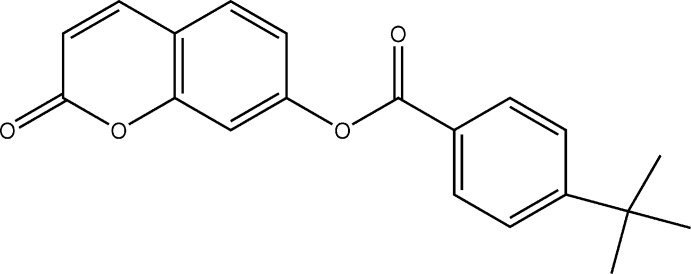


## Structural commentary   

**Figure 1:**
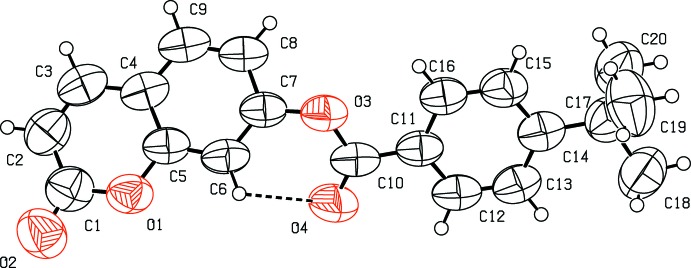
The mol­ecular structure of the title compound and the atomic numbering scheme. Displacement ellipsoids are drawn at the 50% probability level. H atoms are shown as spheres of arbitrary radius. The intra­molecular hydrogen bond is indicated by dashed lines.

The mol­ecular structure of the title coumarin derivative, (I), is illustrated in Fig. 1 ▸. An *S*(6) ring motif arises from an intra­molecular C6—H6⋯O4 hydrogen bond, and generates a pseudo-tricyclic ring system (Fig. 1 ▸). The coumarin ring system is planar [r.m.s deviation = 0.016 Å] and is oriented at an acute angle of 33.10 (12)° with respect to the C11–C16 benzene ring while the pseudo-six-membered ring makes dihedral angles of 27.34 (11) and 13.98 (13)°, respectively, with the coumarin ring system and the benzene ring. An inspection of the bond lengths shows that there is a slight asymmetry of the electronic distribution around the pyrone ring: the C3—C2 [1.338 (5) Å] and C2—C1 [1.426 (5) Å] bond lengths are shorter and longer, respectively, than those excepted for a C_ar_—C_ar_ bond. This suggests that the electron density is preferentially located in the C2—C3 bond of the pyrone ring, as seen in other coumarin derivatives (Gomes *et al.*, 2016 ▸; Ziki *et al.*, 2016 ▸).

## Supra­molecular features   

**Figure 2:**
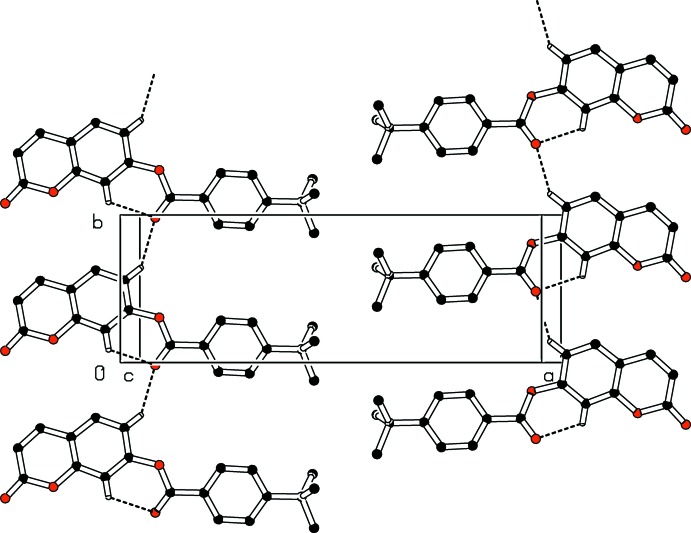
Part of the crystal packing of the title compound showing the formation of an infinite *C*(6) chain along the *b-*axis direction. Dashed lines indicate hydrogen bonds. H atoms not involved in hydrogen-bonding inter­actions have been omitted for clarity.

**Figure 3:**
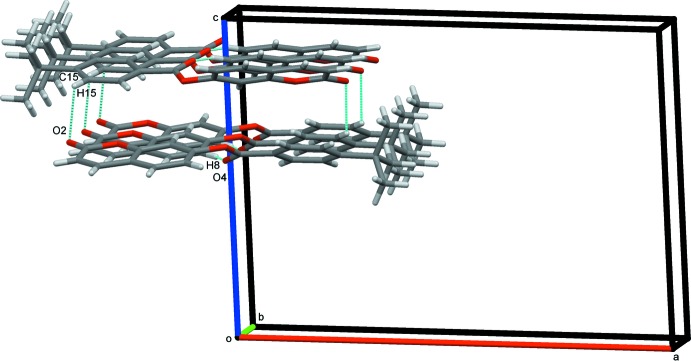
Crystal packing of (I) showing adjacent pairs of mol­ecules along the *b* axis

**Figure 4:**
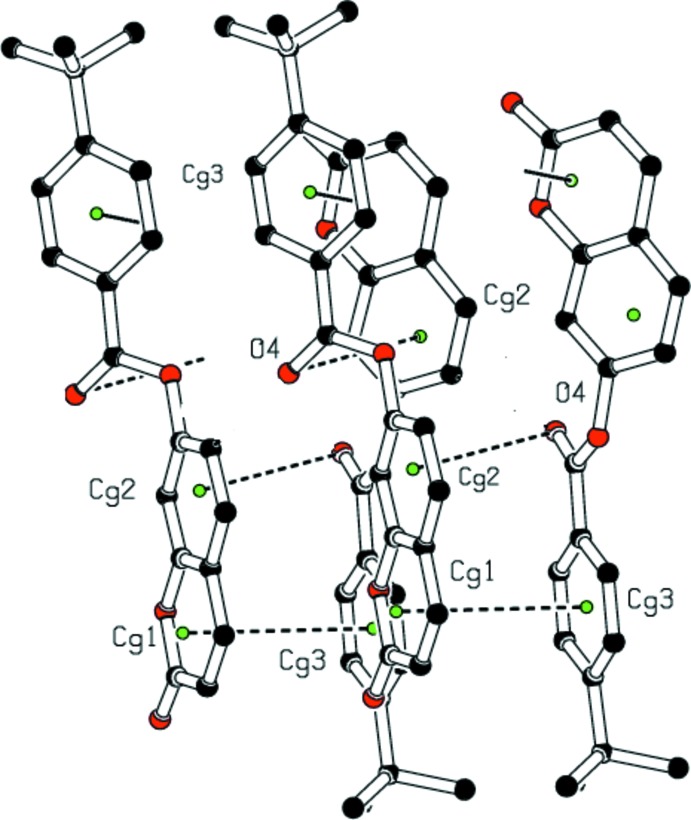
A view of the crystal packing, showing H⋯H contacts, C10=O4⋯π and π–π stacking inter­actions (dashed lines). The green dots are ring centroids. H atoms not involved in H⋯H inter­actions have been omitted for clarity.

In the crystal, two types of inter­molecular hydrogen-bonding inter­actions are present (Table 1 ▸). The C8—H8⋯O4 hydrogen bonds link mol­ecules into infinite chains along the [010] direction (Fig. 2 ▸) while the C15—H15⋯O2 hydrogen-bonding inter­actions generate chains extending along the *c*-axis direction, as shown in Fig. 3 ▸. In addition, a close contact [H2⋯H19*B*(−*x*, −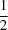 + *y*, 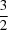 − *z*) = 2.38 Å] is found at a distance shorter than the sum of the van der Waals radii. An unusual C10=O4⋯π inter­action [O4⋯*Cg*2(−*x*, 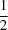 + *y*, 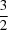 − *z*) = 3.760 (3) Å, where *Cg*2 is the centroid of the C4–C9 benzene ring], is also present. The resulting supra­molecular aggregation is completed by the presence of π–π stacking (Fig. 4 ▸) between the pyrone and benzene rings with centroid–centroid distances [*Cg*1⋯*Cg*3(−*x*, −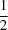 + *y*, 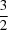 − *z*) = 3.7035 (18) and *Cg*3⋯*Cg*1 (−*x*, 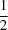 + *y*, 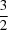 − *z*) = 3.7034 (18) Å, where *Cg*1 and *Cg*3 are the centroids of the pyrone and the C11–C16 benzene rings, respectively] that are less than 3.8 Å, the maximum regarded as suitable for an effective π–π inter­action (Janiak, 2000 ▸). In these inter­actions, the perpendicular distances of *Cg*1 on ring 3 are 3.6144 (13) and 3.6143 (13) Å, respectively, and the distances between *Cg*1 and a perpendicular projection of *C*g3 on ring 1 (slippage) are 0.726 and 0.807Å, respectively.

## Database survey   

A CSD search (Web CSD version 5.39; March 9, 2018; Groom *et al.*, 2016 ▸) found five coumarin ester structures with substituents at the 7 positions (Ramasubbu *et al.*,1982 ▸; Gnanaguru *et al.*, 1985 ▸; Parveen *et al.*, 2011 ▸; Ji *et al.*, 2014 ▸, 2017 ▸). In these structures and those of *meta*-substituted coumarin esters (Abou *et al.*, 2012 ▸, 2013 ▸; Bibila Mayaya Bisseyou *et al.*, 2013 ▸; Yu *et al.*, 2014 ▸; Gomes *et al.*, 2016 ▸; Ziki *et al.*, 2016 ▸, 2017 ▸), the pyrone rings all show three long (in the range 1.37–1.46 Å) and one short (1.32–1.34 Å) C—C distances, suggesting that the electronic density is preferentially located in the short C—C bond at the pyrone ring. This pattern is clearly repeated here with C2—C3 = 1.338 (5) Å while C1—C2 = 1.426 (5), C3—C4 = 1.436 (5) and C4—C5 = 1.375 (4) Å.

## Hirshfeld surface analysis   

**Figure 5:**
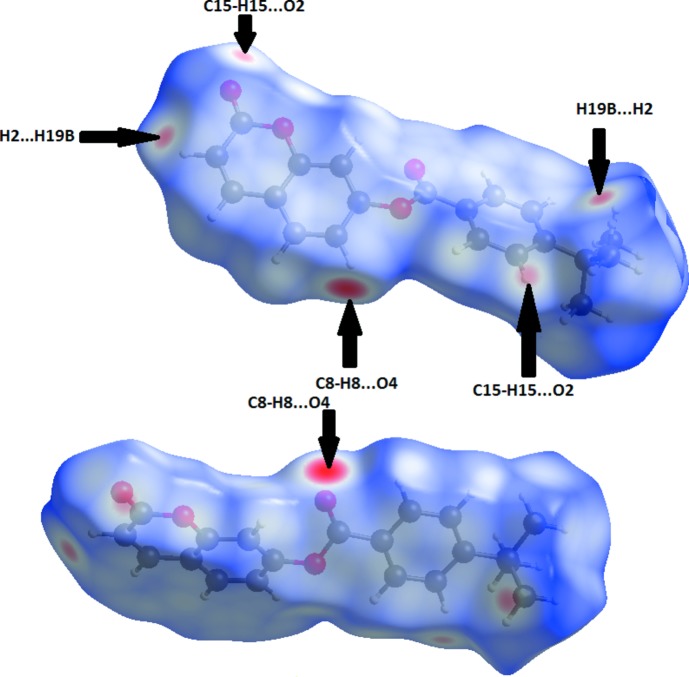
A View of the Hirshfeld surfaces with the three-dimensional *d*_norm_ surfaces mapped over a fixed colour scale of −0.39 (red) to 1.4 Å (blue) for compound (I).

**Figure 6:**
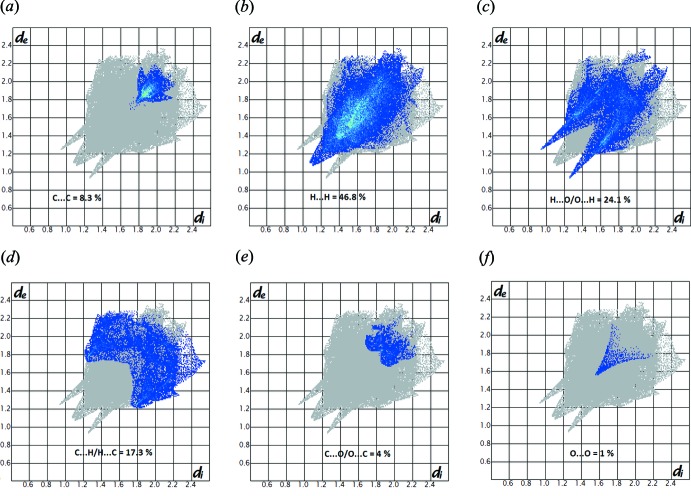
Decomposed two-dimensional fingerprint plots for compound (I). Various close contacts and their relative contributions are indicated.

Mol­ecular Hirshfeld surfaces of 2-oxo-2*H*-chromen-7-yl 4-*tert*-butyl­benzoate, (I), were calculated using a standard (high) surface resolution, and with the three-dimensional *d*_norm_ surfaces mapped over a fixed colour scale of −0.39 (red) to 1.4 Å (blue) with the program *CrystalExplorer 3.1* (Wolff *et al.*, 2012 ▸). The analysis of inter­molecular inter­actions through the mapping of *d*_norm_ is accomplished by considering the contact distances *d*_i_ and *d*_e_ from the Hirshfeld surface to the nearest atom inside and outside, respectively. In (I), the surface mapped over *d*_norm_ highlights six red spots showing distances shorter than the sum of the van der Waals radii. These dominant inter­actions correspond to inter­molecular C—H⋯O hydrogen bonds, O⋯π and π–π stacking inter­actions between the surface and the neighbouring environment. The mapping also shows white spots with distances equal to the sum of the van der Waals radii and blue regions with distances longer than the sum of the van der Waals radii. The surfaces are transparent to allow visualization of the mol­ecule (Fig. 5 ▸). Furthermore, the two-dimensional fingerprint plots (FP) in Fig. 6 ▸ highlight particular close contacts of atom pairs and the contributions from different contacts are provided. The red spots in the middle of the surface appearing near *d*_e_ = *d*_i_ ≃ 1.8–2 Å correspond to close C⋯C inter­planar contacts. These contacts, which comprise 8.3% of the total Hirshfeld surface area, relate to π–π inter­actions (Fig. 6 ▸ *a*), as shown by the X-ray study. The most significant contribution to the Hirshfeld surface (46.8%) is from H⋯H contacts, which appear in the central region of the FP (Fig. 6 ▸ *b*). H⋯O/O⋯H inter­actions with a 24.1% contribution appear as blue spikes in Fig. 6 ▸ *c* and show the presence of O⋯H contacts, whereas the C⋯H/H⋯C plot (17.3%) gives information about inter­molecular hydrogen bonds (Fig. 6 ▸ *d*). Other visible spots in the Hirshfeld surfaces show C⋯O/O⋯C and O⋯O contacts, which contribute only 4.0 and 1.0%, respectively (Fig. 6 ▸ *e* and 6*f*).

## Theoretical calculations   

The geometry optimization of compound (I) was performed using the density functional theory (DFT) method with a 6-311^++^G(d,p) basis set. The crystal structure in the solid state was used as the starting structure for the calculations. The DFT calculations are performed with the *GAUSSIAN09* program package (Frisch *et al.*, 2013 ▸). The resulting geometrical parameters are compared with those obtained from the X-ray crystallographic study. An analysis of the computational bond lengths and bond angles and comparison with the crystallographic results shows a good agreement between them, with a root-mean-square deviation of 0.017 Å for bond lengths and 0.97° for bond angles (see Supplementary Tables S1 and S2). In addition, an inspection of the calculated torsion angles shows that the coumarin ring system and the benzene (C11–C16) ring are planar (Supplementary Table S3), which is in good agreement with the crystallographic prevision, although the calculated C10—O3—C7—C8 torsion angle between them (129.1°) is somewhat lower than the observed value [141.3 (3)°].

## Synthesis and crystallization   

To a solution of 4-*tert*-butyl­benzoyl chloride (6.17 mmol; 1.3 g) in dry tetra­hydro­furan (30 to 40 ml), was added dry tri­methyl­amine (2.6 ml; 3 molar equivalents) and 7-hy­droxy­coumarin (6.17 mmol; 1g) in small portions over 30 min. The mixture was then refluxed for four h and poured into 40 ml of chloro­form. The solution was acidified with diluted hydro­chloric acid until the pH was 2–3. The organic layer was extracted, washed with water to neutrality, dried over MgSO_4_ and the solvent removed. The resulting precipitate (crude product) was filtered off with suction, washed with petroleum ether and recrystallized from chloro­form. Colourless crystals of the title compound were obtained in a good yield: 90%; m.p. 406–408 K.

## Refinement   

Crystal data, data collection and structure refinement details are summarized in Table 2 ▸. H atoms were placed in calculated positions [C—H = 0.93 (aromatic) or 0.96 Å (methyl group)] and refined using a riding-model approximation with *U*_iso_(H) constrained to 1.2 (aromatic) or 1.5 (meth­yl) times *U*eq(C) of the respective parent atom.

## Supplementary Material

Crystal structure: contains datablock(s) I. DOI: 10.1107/S2056989018004188/sj5549sup1.cif

Structure factors: contains datablock(s) I. DOI: 10.1107/S2056989018004188/sj5549Isup2.hkl

Supporting information file. DOI: 10.1107/S2056989018004188/sj5549Isup3.cml

CCDC reference: 1828991

Additional supporting information:  crystallographic information; 3D view; checkCIF report

## Figures and Tables

**Table 1:** Hydrogen-bond geometry (Å, °) *Cg*2 is the centroid of the C4–C9 ring.

*D*—H⋯*A*	*D*—H	H⋯*A*	*D*⋯*A*	*D*—H⋯*A*
C8—H8⋯O4^i^	0.93	2.32	3.114 (4)	144
C15—H15⋯O2^ii^	0.93	2.65	3.310 (4)	128
C6—H6⋯O4	0.93	2.41	2.813 (4)	106
C10—O4⋯*Cg*2^iii^	1.18 (1)	3.76 (1)	3.560 (3)	71 (1)

Symmetry codes: (i) 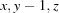; (ii) 
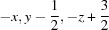
; (iii) 
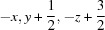
.

**Table 2:** Experimental details

Crystal data	
Chemical formula	C_20_H_18_O_4_
*M*_r_	322.34
Crystal system, space group	Monoclinic, *P*2_1_/*c*
Temperature (K)	298
*a*, *b*, *c* (Å)	18.684 (2), 6.5431 (5), 13.6688 (14)
β (°)	93.627 (11)
*V* (Å^3^)	1667.7 (3)
*Z*	4
Radiation type	Cu *K*α
μ (mm^−1^)	0.73
Crystal size (mm)	0.40 × 0.12 × 0.04

Data collection	
Diffractometer	Rigaku Oxford Diffraction SuperNova, Dual, Cu at zero, Atlas S2
Absorption correction	Multi-scan (*CrysAlis PRO*; Rigaku OD, 2015 ▸)
*T*_min_, *T*_max_	0.714, 1.000
No. of measured, independent and observed [*I* > 2σ(*I*)] reflections	9647, 3005, 1710
*R*_int_	0.035
(sin θ/λ)_max_ (Å^−1^)	0.600

Refinement	
*R*[*F* ^2^ > 2σ(*F* ^2^)], *wR*(*F* ^2^), *S*	0.057, 0.202, 1.01
No. of reflections	3005
No. of parameters	217
H-atom treatment	H-atom parameters constrained
Δρ_max_, Δρ_min_ (e Å^−3^)	0.14, −0.13

Computer programs: *CrysAlis PRO* (Rigaku OD, 2015 ▸), *SIR2014* (Burla *et al.*, 2015 ▸), *PLATON* (Spek, 2009 ▸), *Mercury* (Macrae *et al.*, 2008 ▸), *SHELXL2014* (Sheldrick, 2015 ▸), *publCIF* (Westrip, 2010 ▸) and *WinGX* (Farrugia, 2012 ▸).

## supplementary crystallographic information

### Crystal data

**Table:**

C_20_H_18_O_4_	*F*(000) = 680
*M**_r_* = 322.34	*D*_x_ = 1.284 Mg m^−^^3^
Monoclinic, *P*2_1_/*c*	Melting point = 406–408 K
Hall symbol: -P 2ybc	Cu *K*α radiation, λ = 1.54184 Å
*a* = 18.684 (2) Å	Cell parameters from 1499 reflections
*b* = 6.5431 (5) Å	θ = 4.7–63.4°
*c* = 13.6688 (14) Å	µ = 0.73 mm^−^^1^
β = 93.627 (11)°	*T* = 298 K
*V* = 1667.7 (3) Å^3^	Prism, colorless
*Z* = 4	0.40 × 0.12 × 0.04 mm

### Data collection

**Table:**

Rigaku Oxford Diffraction SuperNova, Dual, Cu at zero, Atlas S2 diffractometer	3005 independent reflections
Radiation source: micro-focus sealed X-ray tube, SuperNova (Cu) X-ray Source	1710 reflections with *I* > 2σ(*I*)
Mirror monochromator	*R*_int_ = 0.035
Detector resolution: 5.3048 pixels mm^-1^	θ_max_ = 67.7°, θ_min_ = 4.7°
ω scans	*h* = −22→19
Absorption correction: multi-scan (CrysAlis PRO; Rigaku OD, 2015)	*k* = −7→7
*T*_min_ = 0.714, *T*_max_ = 1.000	*l* = −15→16
9647 measured reflections	

### Refinement

**Table:**

Refinement on *F*^2^	Primary atom site location: structure-invariant direct methods
Least-squares matrix: full	Secondary atom site location: difference Fourier map
*R*[*F*^2^ > 2σ(*F*^2^)] = 0.057	Hydrogen site location: inferred from neighbouring sites
*wR*(*F*^2^) = 0.202	H-atom parameters constrained
*S* = 1.01	*w* = 1/[σ^2^(*F*_o_^2^) + (0.0981*P*)^2^] where *P* = (*F*_o_^2^ + 2*F*_c_^2^)/3
3005 reflections	(Δ/σ)_max_ < 0.001
217 parameters	Δρ_max_ = 0.14 e Å^−^^3^
0 restraints	Δρ_min_ = −0.13 e Å^−^^3^
72 constraints	

### Special details

**Table:**

Geometry. All esds (except the esd in the dihedral angle between two l.s. planes) are estimated using the full covariance matrix. The cell esds are taken into account individually in the estimation of esds in distances, angles and torsion angles; correlations between esds in cell parameters are only used when they are defined by crystal symmetry. An approximate (isotropic) treatment of cell esds is used for estimating esds involving l.s. planes.

### Fractional atomic coordinates and isotropic or equivalent isotropic displacement parameters (Å^2^)

**Table:**

	*x*	*y*	*z*	*U*_iso_*/*U*_eq_	
O3	0.05338 (12)	0.1955 (3)	0.65709 (16)	0.0967 (6)	
O1	−0.19810 (13)	0.3433 (3)	0.65369 (16)	0.1016 (7)	
O4	0.04152 (14)	0.5177 (3)	0.6031 (2)	0.1233 (9)	
C7	−0.01924 (18)	0.1455 (4)	0.64140 (19)	0.0869 (8)	
C4	−0.15790 (19)	0.0068 (4)	0.61057 (19)	0.0905 (8)	
C6	−0.07476 (18)	0.2783 (4)	0.6568 (2)	0.0904 (8)	
H6	−0.0662	0.4119	0.6776	0.108*	
C11	0.15865 (18)	0.3838 (4)	0.63538 (19)	0.0860 (8)	
C5	−0.14356 (18)	0.2044 (4)	0.63979 (19)	0.0869 (8)	
C9	−0.1001 (2)	−0.1235 (4)	0.5982 (2)	0.0974 (9)	
H9	−0.1086	−0.2586	0.5797	0.117*	
C10	0.0789 (2)	0.3799 (4)	0.6300 (2)	0.0907 (8)	
C12	0.1920 (2)	0.5629 (4)	0.6107 (2)	0.0974 (9)	
H12	0.1645	0.6774	0.5935	0.117*	
C14	0.3083 (2)	0.4064 (5)	0.6350 (2)	0.0971 (9)	
C8	−0.0311 (2)	−0.0549 (4)	0.6129 (2)	0.0933 (8)	
H8	0.0073	−0.1419	0.6038	0.112*	
C3	−0.2317 (2)	−0.0532 (5)	0.5957 (2)	0.1066 (10)	
H3	−0.2432	−0.1866	0.5774	0.128*	
O2	−0.31255 (16)	0.4209 (5)	0.6509 (2)	0.1421 (11)	
C13	0.2657 (2)	0.5738 (5)	0.6111 (2)	0.1024 (10)	
H13	0.2872	0.6965	0.5950	0.123*	
C16	0.20033 (19)	0.2163 (4)	0.6614 (2)	0.0957 (9)	
H16	0.1788	0.0951	0.6794	0.115*	
C15	0.2743 (2)	0.2284 (5)	0.6607 (2)	0.1034 (10)	
H15	0.3018	0.1140	0.6781	0.124*	
C1	−0.2694 (2)	0.2881 (6)	0.6378 (3)	0.1124 (10)	
C2	−0.2839 (2)	0.0826 (6)	0.6080 (3)	0.1137 (11)	
H2	−0.3314	0.0415	0.5968	0.136*	
C17	0.3902 (2)	0.4102 (6)	0.6331 (3)	0.1112 (10)	
C18	0.4185 (3)	0.6214 (7)	0.6053 (4)	0.1529 (18)	
H18A	0.3973	0.6606	0.5423	0.229*	
H18B	0.4062	0.7200	0.6535	0.229*	
H18C	0.4697	0.6157	0.6028	0.229*	
C19	0.4251 (3)	0.3551 (9)	0.7329 (4)	0.170 (2)	
H19A	0.4763	0.3583	0.7302	0.256*	
H19B	0.4107	0.4518	0.7807	0.256*	
H19C	0.4103	0.2205	0.7508	0.256*	
C20	0.4126 (3)	0.2572 (8)	0.5546 (4)	0.166 (2)	
H20A	0.3901	0.2942	0.4920	0.249*	
H20B	0.4638	0.2604	0.5512	0.249*	
H20C	0.3979	0.1219	0.5717	0.249*	

### Atomic displacement parameters (Å^2^)

**Table:**

	*U*^11^	*U*^22^	*U*^33^	*U*^12^	*U*^13^	*U*^23^
O3	0.1181 (17)	0.0720 (10)	0.0991 (14)	0.0106 (10)	−0.0014 (11)	0.0063 (9)
O1	0.1168 (17)	0.0875 (13)	0.0988 (14)	0.0074 (11)	−0.0057 (12)	−0.0189 (10)
O4	0.129 (2)	0.0812 (13)	0.160 (2)	0.0214 (13)	0.0119 (16)	0.0276 (13)
C7	0.119 (2)	0.0684 (13)	0.0726 (15)	0.0089 (14)	−0.0013 (14)	0.0037 (10)
C4	0.132 (2)	0.0739 (14)	0.0647 (14)	−0.0064 (15)	0.0000 (14)	0.0019 (10)
C6	0.128 (2)	0.0655 (13)	0.0764 (15)	0.0065 (14)	−0.0047 (14)	−0.0088 (11)
C11	0.120 (2)	0.0690 (13)	0.0681 (14)	0.0067 (13)	−0.0017 (14)	−0.0029 (10)
C5	0.121 (2)	0.0732 (13)	0.0660 (14)	0.0058 (15)	−0.0021 (13)	−0.0042 (11)
C9	0.150 (3)	0.0642 (13)	0.0779 (16)	0.0016 (16)	0.0037 (17)	0.0010 (11)
C10	0.133 (3)	0.0646 (13)	0.0730 (15)	0.0115 (14)	−0.0011 (15)	−0.0031 (11)
C12	0.133 (3)	0.0688 (14)	0.0890 (18)	0.0092 (15)	−0.0019 (17)	0.0035 (12)
C14	0.124 (3)	0.0839 (17)	0.0813 (17)	−0.0018 (16)	−0.0056 (16)	−0.0001 (13)
C8	0.130 (3)	0.0681 (14)	0.0815 (16)	0.0106 (15)	0.0045 (16)	0.0028 (12)
C3	0.143 (3)	0.0864 (18)	0.0893 (19)	−0.0144 (19)	0.0001 (19)	−0.0027 (14)
O2	0.126 (2)	0.149 (2)	0.149 (2)	0.0183 (18)	−0.0063 (17)	−0.0434 (19)
C13	0.132 (3)	0.0772 (16)	0.097 (2)	−0.0074 (16)	0.0000 (18)	0.0070 (14)
C16	0.121 (3)	0.0740 (15)	0.0915 (19)	0.0033 (15)	0.0038 (16)	0.0098 (13)
C15	0.123 (3)	0.0817 (17)	0.104 (2)	0.0078 (16)	−0.0040 (18)	0.0128 (15)
C1	0.127 (3)	0.114 (2)	0.095 (2)	0.003 (2)	−0.0028 (19)	−0.0183 (18)
C2	0.123 (3)	0.115 (3)	0.102 (2)	−0.016 (2)	0.002 (2)	−0.0086 (19)
C17	0.115 (3)	0.109 (2)	0.108 (2)	−0.0068 (19)	−0.0027 (19)	−0.0005 (18)
C18	0.137 (4)	0.133 (3)	0.185 (5)	−0.031 (3)	−0.019 (3)	0.017 (3)
C19	0.119 (3)	0.246 (6)	0.143 (4)	0.016 (3)	−0.012 (3)	0.050 (4)
C20	0.140 (4)	0.166 (4)	0.198 (5)	−0.026 (3)	0.052 (4)	−0.048 (4)

### Geometric parameters (Å, º)

**Table:**

O3—C10	1.357 (3)	C8—H8	0.9300
O3—C7	1.399 (4)	C3—C2	1.338 (5)
O1—C1	1.383 (4)	C3—H3	0.9300
O1—C5	1.387 (3)	O2—C1	1.206 (4)
O4—C10	1.184 (3)	C13—H13	0.9300
C7—C6	1.379 (4)	C16—C15	1.385 (5)
C7—C8	1.382 (4)	C16—H16	0.9300
C4—C5	1.375 (4)	C15—H15	0.9300
C4—C9	1.395 (5)	C1—C2	1.426 (5)
C4—C3	1.436 (5)	C2—H2	0.9300
C6—C5	1.379 (4)	C17—C19	1.517 (5)
C6—H6	0.9300	C17—C18	1.536 (5)
C11—C16	1.378 (4)	C17—C20	1.545 (5)
C11—C12	1.379 (4)	C18—H18A	0.9600
C11—C10	1.488 (5)	C18—H18B	0.9600
C9—C8	1.369 (5)	C18—H18C	0.9600
C9—H9	0.9300	C19—H19A	0.9600
C12—C13	1.380 (5)	C19—H19B	0.9600
C12—H12	0.9300	C19—H19C	0.9600
C14—C13	1.381 (4)	C20—H20A	0.9600
C14—C15	1.383 (4)	C20—H20B	0.9600
C14—C17	1.531 (5)	C20—H20C	0.9600
			
C10—O3—C7	121.3 (2)	C14—C13—H13	119.3
C1—O1—C5	121.1 (3)	C11—C16—C15	120.1 (3)
C6—C7—C8	122.2 (3)	C11—C16—H16	120.0
C6—C7—O3	124.1 (3)	C15—C16—H16	120.0
C8—C7—O3	113.7 (3)	C14—C15—C16	121.7 (3)
C5—C4—C9	118.2 (3)	C14—C15—H15	119.2
C5—C4—C3	117.8 (3)	C16—C15—H15	119.2
C9—C4—C3	124.0 (3)	O2—C1—O1	115.8 (3)
C5—C6—C7	117.1 (3)	O2—C1—C2	127.2 (4)
C5—C6—H6	121.5	O1—C1—C2	117.0 (4)
C7—C6—H6	121.5	C3—C2—C1	122.3 (4)
C16—C11—C12	118.8 (3)	C3—C2—H2	118.8
C16—C11—C10	123.2 (3)	C1—C2—H2	118.8
C12—C11—C10	118.0 (3)	C19—C17—C14	110.7 (3)
C4—C5—C6	122.8 (3)	C19—C17—C18	107.5 (4)
C4—C5—O1	121.6 (3)	C14—C17—C18	112.2 (3)
C6—C5—O1	115.6 (2)	C19—C17—C20	110.6 (4)
C8—C9—C4	120.8 (3)	C14—C17—C20	108.4 (3)
C8—C9—H9	119.6	C18—C17—C20	107.3 (4)
C4—C9—H9	119.6	C17—C18—H18A	109.5
O4—C10—O3	123.5 (3)	C17—C18—H18B	109.5
O4—C10—C11	124.8 (3)	H18A—C18—H18B	109.5
O3—C10—C11	111.7 (2)	C17—C18—H18C	109.5
C11—C12—C13	120.6 (3)	H18A—C18—H18C	109.5
C11—C12—H12	119.7	H18B—C18—H18C	109.5
C13—C12—H12	119.7	C17—C19—H19A	109.5
C13—C14—C15	117.4 (4)	C17—C19—H19B	109.5
C13—C14—C17	123.0 (3)	H19A—C19—H19B	109.5
C15—C14—C17	119.6 (3)	C17—C19—H19C	109.5
C9—C8—C7	119.0 (3)	H19A—C19—H19C	109.5
C9—C8—H8	120.5	H19B—C19—H19C	109.5
C7—C8—H8	120.5	C17—C20—H20A	109.5
C2—C3—C4	120.1 (3)	C17—C20—H20B	109.5
C2—C3—H3	119.9	H20A—C20—H20B	109.5
C4—C3—H3	119.9	C17—C20—H20C	109.5
C12—C13—C14	121.4 (3)	H20A—C20—H20C	109.5
C12—C13—H13	119.3	H20B—C20—H20C	109.5
			
C10—O3—C7—C6	−41.5 (4)	C6—C7—C8—C9	0.8 (4)
C10—O3—C7—C8	141.3 (3)	O3—C7—C8—C9	178.1 (3)
C8—C7—C6—C5	−1.7 (4)	C5—C4—C3—C2	1.5 (5)
O3—C7—C6—C5	−178.7 (2)	C9—C4—C3—C2	−179.2 (3)
C9—C4—C5—C6	0.3 (4)	C11—C12—C13—C14	0.8 (5)
C3—C4—C5—C6	179.7 (3)	C15—C14—C13—C12	−1.5 (5)
C9—C4—C5—O1	180.0 (2)	C17—C14—C13—C12	177.9 (3)
C3—C4—C5—O1	−0.6 (4)	C12—C11—C16—C15	−1.1 (5)
C7—C6—C5—C4	1.2 (4)	C10—C11—C16—C15	177.2 (3)
C7—C6—C5—O1	−178.5 (2)	C13—C14—C15—C16	0.9 (5)
C1—O1—C5—C4	−0.5 (4)	C17—C14—C15—C16	−178.5 (3)
C1—O1—C5—C6	179.2 (3)	C11—C16—C15—C14	0.4 (5)
C5—C4—C9—C8	−1.3 (4)	C5—O1—C1—O2	−179.1 (3)
C3—C4—C9—C8	179.4 (3)	C5—O1—C1—C2	0.8 (5)
C7—O3—C10—O4	9.6 (4)	C4—C3—C2—C1	−1.2 (5)
C7—O3—C10—C11	−168.7 (2)	O2—C1—C2—C3	180.0 (4)
C16—C11—C10—O4	−175.9 (3)	O1—C1—C2—C3	0.1 (6)
C12—C11—C10—O4	2.4 (5)	C13—C14—C17—C19	122.1 (4)
C16—C11—C10—O3	2.3 (4)	C15—C14—C17—C19	−58.5 (5)
C12—C11—C10—O3	−179.4 (2)	C13—C14—C17—C18	1.9 (5)
C16—C11—C12—C13	0.6 (4)	C15—C14—C17—C18	−178.7 (4)
C10—C11—C12—C13	−177.8 (3)	C13—C14—C17—C20	−116.5 (4)
C4—C9—C8—C7	0.7 (4)	C15—C14—C17—C20	62.9 (4)

### Hydrogen-bond geometry (Å, º)

Cg2 is the centroid of the C4–C9 ring.

**Table:**

*D*—H···*A*	*D*—H	H···*A*	*D*···*A*	*D*—H···*A*
C8—H8···O4^i^	0.93	2.32	3.114 (4)	144
C15—H15···O2^ii^	0.93	2.65	3.310 (4)	128
C6—H6···O4	0.93	2.41	2.813 (4)	106
C10—O4···*Cg*2^iii^	1.18 (1)	3.76 (1)	3.560 (3)	71 (1)

Symmetry codes: (i) *x*, *y*−1, *z*; (ii) −*x*, *y*−1/2, −*z*+3/2; (iii) −*x*, *y*+1/2, −*z*+3/2.

### S1

Experimental and calculated bond lengths (Å)

**Table:**

Bond	X-ray	6-311^++^G(d,p)
O3—C10	1.357 (3)	1.381
O3—C7	1.399 (4)	1.387
O1—C1	1.383 (4)	1.399
O1—C5	1.387 (3)	1.363
O4—C10	1.184 (3)	1.203
C7—C6	1.379 (4)	1.387
C7—C8	1.382 (4)	1.399
C4—C5	1.375 (4)	1.406
C4—C9	1.395 (5)	1.405
C4—C3	1.436 (5)	1.438
C6—C5	1.379 (4)	1.392
C11—C16	1.378 (4)	1.401
C11—C12	1.379 (4)	1.397
C11—C10	1.488 (5)	1.482
C9—C8	1.369 (5)	1.383
C12—C13	1.380 (5)	1.391
C14—C13	1.381 (4)	1.401
C14—C15	1.383 (4)	1.405
C14—C17	1.531 (5)	1.537
C3—C2	1.338 (5)	1.350
O2—C1	1.206 (4)	1.202
C16—C15	1.385 (5)	1.388
C1—C2	1.426 (5)	1.457
C17—C19	1.517 (5)	1.547
C17—C18	1.536 (5)	1.540
C17—C20	1.545 (5)	1.547

### S2

Experimental and calculated bond angles (°)

**Table:**

Bond angle	X-ray	6-311^++^G(d,p)
C10—O3—C7	121.3 (2)	120.5
C1—O1—C5	121.1 (3)	122.9
C6—C7—C8	122.2 (3)	121.8
C6—C7—O3	124.1 (3)	121.7
C8—C7—O3	113.7 (3)	116.5
C5—C4—C9	118.2 (3)	118.3
C5—C4—C3	117.8 (3)	117.5
C9—C4—C3	124.0 (3)	124.3
C5—C6—C7	117.1 (3)	118.2
C16—C11—C12	118.8 (3)	118.9
C16—C11—C10	123.2 (3)	123.1
C12—C11—C10	118.0 (3)	118.0
C4—C5—C6	122.8 (3)	121.7
C4—C5—O1	121.6 (3)	121.2
C6—C5—O1	115.6 (2)	117.0
C8—C9—C4	120.8 (3)	120.8
O4—C10—O3	123.5 (3)	123.0
O4—C10—C11	124.8 (3)	125.7
O3—C10—C11	111.7 (2)	111.4
C11—C12—C13	120.6 (3)	120.5
C13—C14—C15	117.4 (4)	117.3
C13—C14—C17	123.0 (3)	122.8
C15—C14—C17	119.6 (3)	119.9
C9—C8—C7	119.0 (3)	119.2
C2—C3—C4	120.1 (3)	120.9
C12—C13—C14	121.4 (3)	121.4
C11—C16—C15	120.1 (3)	120.1
C14—C15—C16	121.7 (3)	121.8
O2—C1—O1	115.8 (3)	117.7
O2—C1—C2	127.2 (4)	126.4
O1—C1—C2	117.0 (4)	115.9
C3—C2—C1	122.3 (4)	121.6
C19—C17—C14	110.7 (3)	109.3
C19—C17—C18	107.5 (4)	108.2
C14—C17—C18	112.2 (3)	112.4
C19—C17—C20	110.6 (4)	109.4
C14—C17—C20	108.4 (3)	109.3
C18—C17—C20	107.3 (4)	108.2

### S3

Experimental and calculated torsion angles (°)

**Table:**

Torsion angle	X-ray	6-311^++^G(d,p)
C10—O3—C7—C6	-41.5 (4)	-54.7
C10—O3—C7—C8	141.3 (3)	129.1
C8—C7—C6—C5	-1.7 (4)	-0.3
O3—C7—C6—C5	-178.7 (2)	-176.3
C9—C4—C5—C6	0.3 (4)	0.1
C3—C4—C5—C6	179.7 (3)	-180.0
C9—C4—C5—O1	180.0 (2)	-179.7
C3—C4—C5—O1	-0.6 (4)	0.3
C7—C6—C5—C4	1.2 (4)	0.2
C7—C6—C5—O1	-178.5 (2)	179.9
C1—O1—C5—C4	-0.5 (4)	-0.0
C1—O1—C5—C6	179.2 (3)	-179.8
C5—C4—C9—C8	-1.3 (4)	-0.2
C3—C4—C9—C8	179.4 (3)	179.9
C7—O3—C10—O4	9.6 (4)	-2.1
C7—O3—C10—C11	-168.7 (2)	178.3
C16—C11—C10—O4	-175.9 (3)	178.9
C12—C11—C10—O4	2.4 (5)	-1.0
C16—C11—C10—O3	2.3 (4)	-1.6
C12—C11—C10—O3	-179.4 (2)	178.6
C16—C11—C12—C13	0.6 (4)	0.1
C10—C11—C12—C13	-177.8 (3)	179.9
C4—C9—C8—C7	0.7 (4)	0.0
C6—C7—C8—C9	0.8 (4)	0.2
O3—C7—C8—C9	178.1 (3)	176.4
C5—C4—C3—C2	1.5 (5)	-0.23
C9—C4—C3—C2	-179.2 (3)	179.7
C11—C12—C13—C14	0.8 (5)	-0.1
C15—C14—C13—C12	-1.5 (5)	0.0
C17—C14—C13—C12	177.9 (3)	-180.0
C12—C11—C16—C15	-1.1 (5)	0.0
C10—C11—C16—C15	177.2 (3)	-179.8
C13—C14—C15—C16	0.9 (5)	0.1
C17—C14—C15—C16	-178.5 (3)	-179.9
C11—C16—C15—C14	0.4 (5)	-0.1
C5—O1—C1—O2	-179.1 (3)	179.7
C5—O1—C1—C2	0.8 (5)	-0.3
C4—C3—C2—C1	-1.2 (5)	-0.1
O2—C1—C2—C3	180.0 (4)	-179.6
O1—C1—C2—C3	0.1 (6)	0.4
C13—C14—C17—C19	122.1 (4)	119.9
C15—C14—C17—C19	-58.5 (5)	-60.1
C13—C14—C17—C18	1.9 (5)	-0.3
C15—C14—C17—C18	-178.7 (4)	179.7
C13—C14—C17—C20	-116.5 (4)	-120.4
C15—C14—C17—C20	62.9 (4)	59.6
